# Ectopic Lymphoid Structures: Powerhouse of Autoimmunity

**DOI:** 10.3389/fimmu.2016.00430

**Published:** 2016-10-17

**Authors:** Elisa Corsiero, Alessandra Nerviani, Michele Bombardieri, Costantino Pitzalis

**Affiliations:** ^1^Centre for Experimental Medicine and Rheumatology, William Harvey Research Institute, Barts and The London School of Medicine and Dentistry, Queen Mary University of London, London, UK

**Keywords:** tertiary lymphoid structures, autoimmunity, lymphoid chemokines, autoimmune diseases, ectopic germinal center

## Abstract

Ectopic lymphoid structures (ELS) often develop at sites of inflammation in target tissues of autoimmune diseases, such as rheumatoid arthritis, Sjögren’s syndrome, multiple sclerosis, myasthenia gravis, and systemic lupus erythematosus. ELS are characterized by the formation of organized T/B cells aggregates, which can acquire follicular dendritic cells network supporting an ectopic germinal center response. In this review, we shall summarize the mechanisms that regulate the formation of ELS in tertiary lymphoid organs, with particular emphasis on the role of lymphoid chemokines in both formation and maintenance of ELS, the role of emerging positive and negative regulators of ELS development and function, including T follicular helper cells and IL-27, respectively. Finally, we shall discuss the main functions of ELS in supporting the affinity maturation, clonal selection, and differentiation of autoreactive B cells contributing to the maintenance and perpetuation of humoral autoimmunity.

## Introduction

It is now well appreciated that ectopic (or tertiary) lymphoid structures (ELS) can develop in target organs of several autoimmune diseases, which are sites of chronic inflammation, although they can also develop in association with cancer, infection, and graft rejection, as previously discussed (1). At particular mucosal sites, ELS can be named based on the site of inflammation where they occur, e.g., inducible bronchus-associated or gut-associated lymphoid tissue or iGALT and iBALT, respectively (2). ELS are characterized by aggregates of T and B cells often showing T/B segregation (i.e., separated areas within the aggregates densely populated by T or B cells only), development of high endothelial venules (HEVs), and, in the majority of cases, follicular dendritic cell (FDC) networks. Compared to lymphoid aggregates forming in secondary lymphoid organs (SLOs), ELS are transient structures that can be triggered by immunization or infection (3) and often resolve after antigen clearance (2). However, in autoimmune diseases, ELS mostly develop in the context of a chronic inflammation, contribute to maintain the disease process, and are often associated with a more severe disease course (1, 4). As we shall discuss later in this review, ELS developing in chronic autoimmune conditions are capable of activating the molecular machinery to sustain *in situ* antibody diversification, isotype switching, B cell differentiation, and oligoclonal expansion, which are in keeping with their ability to function as active ectopic germinal centers (GCs), which can also support the production of autoreactive plasma cells at the local site of inflammation.

The biological events that bring to ELS formation in disease tissues show numerous similarities with the signaling pathways involved in secondary lymphoid tissue organogenesis; nevertheless, there are site-specific differences, particularly regarding the cellular sources of the key factors regulating lymphoid neogenesis, which depend, at least in part, on the nature of the site of inflammation (1, 2).

In this review, we shall first focus on the mechanisms regulating ELS formation and functions, including the well-established role of lymphoid chemokines and lymphotoxins (LTs) in ELS formation, together with the emerging importance of cytokines as positive (i.e., IL-21, IL-22) and negative (i.e., IL-27) regulators in ELS development, maintenance, and function. In the second part of this review, we shall discuss the evidence supporting the concept that ELS in autoimmune diseases contribute to the perpetuation and spreading of autoimmunity *via* the differentiation autoantibody-producing cells selected for disease-specific antigens within ectopic GCs. We will mostly focus on rheumatic autoimmune diseases, such as rheumatoid arthritis (RA) and Sjögren’s syndrome (SS), but we will also refer to other organ-specific autoimmune conditions to highlight differences in the antigen-driven process underlying ELS formation.

## Development and Organization of ELS: The Role of Lymphotoxins and Lymphoid Chemokines

The signals regulating ELS formation and perpetuation, mostly referred as lymphoid neogenesis, largely overlap with those regulating the same events in SLOs during embryonic life, known as lymphoid organogenesis, but with notable differences in the cellular sources of these factors in ELS development (5). Either in SLOs and ELS formation and perpetuation, the chemoattractant signaling pathway involves several homeostatic lymphoid chemokines, such as CXCL13, CCL19, CCL21, and CXCL12, and their specific receptors CXCR5 (for CXCL13), CCR7 (for CCL19 and CCL21), and CXCR4 (for CXCL12). In classic models of lymphoid organogenesis, the interaction between hematopoietic lymphoid tissue inducer cells (CD3^−^CD4^+^IL-7Ra^+^RANK^+^) and VCAM-1 + ICAM-1 + LTBR^+^ mesenchymal organizer cells drives the establishment of a LTα1β2 (also known as LTβ)/lymphoid chemokine feedback loop, which is required for SLOs development including early B/T cell clustering and segregation as well as the differentiation of HEVs, as reviewed extensively elsewhere (1, 6, 7). Conversely, the early stages of lymphoid neogenesis in adult life are not fully understood, although recent evidence suggests an important role for inflammatory cytokines, such as IL-22 and IL-17, as early contributors in ELS formation, as discussed later. Regardless, once ELS are established, additional and/or alternative cell types can express lymphoid chemokines and LTs during chronic inflammation in autoimmune conditions. In ELS, myofibroblast-like stromal cells support the production of CCL21 around HEVs in the T cell-rich area of the lymphoid aggregate, whereas CXCL13 can be produced by infiltrating cells (i.e. CD14^+^ inflammatory monocytes, CD68^+^ macrophages, and memory CD3^+^CD4^+^ T cells) but also resident tissue cells such as activated stromal and epithelial cells (6). Therefore, it is believed that the immune cells recruited at the site of inflammation, in cross-talk with resident cells which are tissue-specific, exert an active role in the initiation of ELS development (2). Another example of the importance of the site-specific inflammatory milieu in ELS development and/or maintenance in autoimmune diseases is represented by TNF-α, which is abundantly expressed in the synovium of RA patients. In this regard, evidence that TNF-blockade can reverse ELS formation in the joints, at least in a subset of patients, would suggest that in some conditions, TNF-α can play a non-redundant role in ELS maintenance over and above LT-β (8).

Once ELS are established, lymphoid chemokines CCL19, CCL21, and CXCL13 are critical for their perpetuation and function by controlling the homing and tissue localization of immune cells subsets, which are crucial in adaptive immune responses. The concomitant presence of CCL19/CCL21 and peripheral node addressin (PNAd-positive) HEVs allows the homing of CCR7^+^ T cells (i.e., naïve and central memory) and mature CCR7^+^ dendritic cells (DCs) from the systemic circulation upon binding to PNAd^+^ HEVs through L-selectin (6, 9). Naïve B cells can also express at lower level CCR7, and together with CXCR4 and CXCR5, they use these receptors to enter ELS from the systemic circulation (6, 10), although B cell positioning into ectopic follicles is mainly controlled by CXCR5 in response to a CXCL13 gradient. B cells can actively contribute to ELS maintenance as they become strong producers of LTβ.

Lymphoid chemokines CXCL13 and CXCL12 are also critical in the function of ELS as ectopic GCs by regulating the shuttling of B cells between the dark and light zones. Inside the GC, CXCL13, mostly produced by FDCs, mainly directs GC B cells to the light zone of the GC where antigen selection occurs. Within the GC, CXCL12, mostly produced by tingible body macrophages in ELS (11), is critically involved in the migration of CXCR4^high^ centroblasts to the dark zone, where somatic hypermutation of the B cell receptor takes place (6). As discussed later in this review, in the target organs of autoimmune diseases, the formation of functional GCs as a result of the lymphoid neogenesis process is critical in the selection and differentiation of autoantibody-producing B cells.

## The Emerging Role of Cytokines as Positive and Negative Regulators of ELS Formation, Perpetuation, and Function

As mentioned earlier, besides the classic LT/lymphoid chemokines feedback loop, there is strong emerging evidence that cytokines produced in the context of the inflammatory process are also critically involved in the lymphoid neogenesis in autoimmune diseases. These include, not exhaustively, IL-17, IL-21, IL-22, IL-23, and TNF (2). For instance, IL-17 produced by a subset of podoplanin-expressing CD4 T cells has been strongly linked with ELS formation in animal models of inducible ELS, and the IL-23/IL-17 axis has been recently associated with ELS formation in RA (12, 13). Because the role of IL-17 in ELS has been recently reviewed extensively (1, 2), here we will focus on the emerging role of positive and negative regulators of ELS formation and function such as IL-21/IL-22 and IL-27, respectively.

Using a model of inducible ELS formation, autoimmunity and exocrine dysfunction resembling SS that we recently developed and which is triggered by local viral infection in the salivary glands of C57BL/6 mice (3), Barone et al. demonstrated that the early production (i.e., within few hours from viral infection) of IL-22, a cytokine belonging to the IL-10 family, by γδT-cells first and by conventional CD4 T cells thereafter, was directly responsible for the induction of CXCL13 by a subset of resident stromal cells expressing gp38. Although IL-22 was able to induce CXCL13 *in vitro* in a LT-β-independent manner, it is yet to be established whether IL-22 is sufficient to induce ELS *in vivo* in the absence of lymphotoxins (12). These findings seem applicable to ELS forming in human autoimmune diseases, as IL-22 has been associated with the formation of inflammatory aggregates both in RA and SS (13, 14).

While IL-22 appears critical in the early phase of ELS development, another cytokine, IL-21, a member of the common cytokine receptor γ chain-binding family, has been shown to play a fundamental role in the function of ELS as ectopic GCs. This cytokine is primarily produced by T follicular helper (Tfh) cells, a highly specialized subset of CD4^+^ memory T cells expressing high amount of CXCR5 (15) and thus able to migrate toward B cell follicles in response to CXCL13 production by FDCs.

In the last decade, Tfh cells have emerged as essential players in the regulation of B cell activation, antibody affinity maturation, and the GC reaction *via* the expression of surface receptors such as inducible T-cell costimulator (ICOS) and programed cell death protein 1 (PD1). They also express the transcription factor B cell lymphoma protein 6 (Bcl-6), which promotes the expression of CXCR5 and represses other T-cell subset-specific transcription factors (16–18). IL-21 is the main soluble factor released by Tfh and binds a receptor complex consisting of the common γ chain and a unique IL-21R. On B cells, IL-21/IL-21R interactions provide potent signaling for B-cell survival, proliferation, and differentiation (19). Indeed, the absence of Tfh cells impairs GC formation and the generation of long-lived plasma cells, resulting in impaired high affinity antibody responses (20).

Among autoimmune diseases, elevated frequencies of Tfh cells in the peripheral blood have been demonstrated in RA, SS, multiple sclerosis (MS), myasthenia gravis (MG), and systemic lupus erythematosus (SLE) (21). Not surprisingly, IL-21 directly correlated with the frequency of Tfh-like cells. Both IL-21 level and number of Tfh-like cells were associated with higher titer of anti-CCP antibodies and disease activity score in RA (22). In the context of ELS, IL-21 and IL-21R expression are upregulated in the synovial tissue of RA patients, whereby IL-21 strictly segregates with the formation of ELS (23–25). Moreover, blocking IL-21/IL-21R in animal models of RA and SS has a beneficial effect on the disease progression (26, 27).

Interestingly, plasticity between Th17 cells (but also Th1 and Th2) with Tfh cells has been observed (28) and may play an important role in autoimmune diseases, including models of experimental MS, whereby Th17 cells crossing the blood–brain barrier can acquire a Tfh-like cells phenotype, thus supporting ELS development and function in the central nervous system (2, 4). However, and of likely relevance to ELS and ectopic GC, Tfh2 and Tfh17, but not Tfh1, are able to secrete IL-21 and induce naïve B cells to secrete class-switched immunoglobulin (Ig) (28).

Together with positive regulators of ELS formation, the existence of cytokines exerting a negative role on lymphoid neogenesis, such as IL-27, has been recently described. IL-27 is a heterodimeric cytokine belonging to the IL-12 family and is composed of EBI3 and IL-27p28 (29). IL-27 signals through a receptor were composed of IL-27Rα and gp130, the latter used also by other cytokines such as IL-6 (29). IL-27 seems to be able to limit antibody production since overexpression of IL-27Rα in the MLR/lpr mouse model of lupus can ameliorate the antibody response (29). Moreover, it has been recently shown that IL27Rα^−/−^ mice developed a more severe form of arthritis after immunization with methylated bovine serum albumin (mBSA), which was also characterized by multiple lymphoid aggregates forming in the inflamed synovial tissue (25). IL-27 can also restrict the expansion of Th17 cells and suppress secretion of IL-17, a cytokine associated also with survival and proliferation of B cells (25, 29, 30). In regard to ELS development, e.g., in human synovium, it has been observed that IL-27 is inversely correlated with the degree of lymphocytic infiltration in the inflamed tissue as well as with the expression of IL-17 and IL-21 at mRNA level (25). However, further experimental and mechanistic data will be required before exploiting the role of IL-27 as a negative regulator of ELS formation and function for therapeutic purposes in autoimmune diseases.

## The Role of ELS as Perpetuators of Autoimmunity in the Target Organs of Autoimmune Diseases

**Figure 1:**
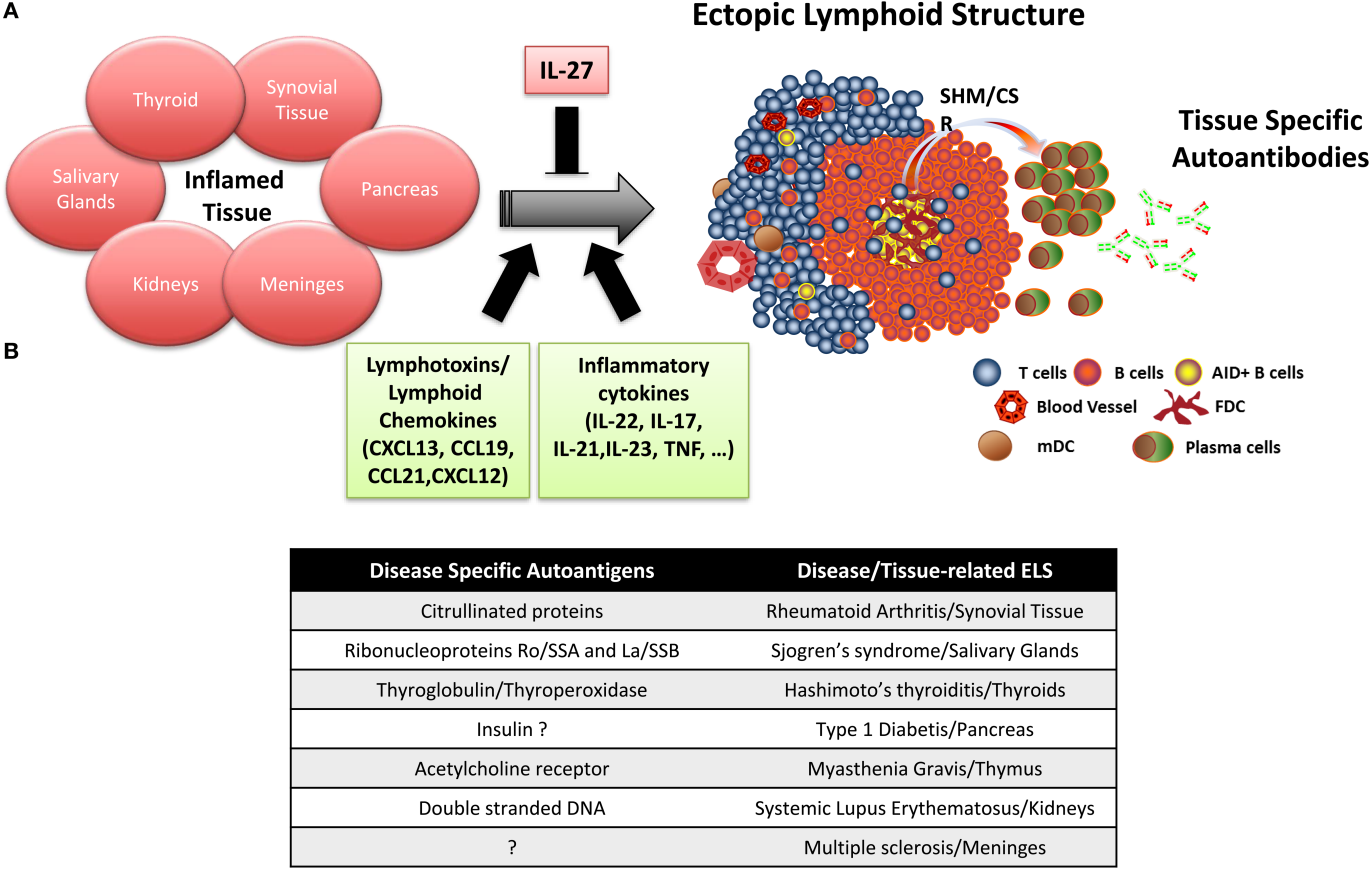
**Ectopic lymphoid structure (ELS) formation in autoimmune disease tissue**. **(A)** Importance of lymphotoxins/lymphoid chemokines and inflammatory cytokines in the formation and maintenance of ELS in the target organs of patients with autoimmune diseases, where ELS preferentially drives the affinity maturation and clonal selection of autoantibodies toward specific autoantigens. **(B)** Summary table listing the main disease-specific autoantigens and the associated autoimmune disease where ELS formation has been observed.

Ectopic lymphoid structures arise in the target organs of patients affected by autoimmune diseases, such as salivary glands in SS (6), synovial tissue in RA (31), kidneys in SLE (32), thymuses in MG (33), meninges in MS (34), and thyroids in Hashimoto’s thyroiditis (35) (Figure 1A). However, for reasons that are currently not clear, the frequency of ELS in these conditions varies significantly, from a minority of patients with SLE to virtually 100% of patients with thyroiditis (36). For instance, in the RA synovium, the immune infiltrates can be arranged into three main microstructural levels of organization including (i) follicular synovitis with ELS (lymphoid pathotype, 40% of RA patients) (31, 37, 38); (ii) diffuse pattern of infiltration with a predominant macrophages component (myeloid pathotype); and (iii) pauci-immune synovitis, characterized by a virtual absence of immune cells (fibroid pathotype) (39). Similarly, around 30–40% of patients with SS show ELS in the affected salivary glands (40, 41), and patients with ELS are significantly more likely to develop B-cell lymphomas of the mucosal-associated lymphoid tissue (MALT-L) (42, 43). In lupus nephritis, B/T cells aggregates can be found in up to 50% of patients in the tubulo-interstitium, but fully organized ectopic GC follicles are detectable in <10% of patients (44). As previously mentioned, ELS in autoimmune conditions are not only structurally reminiscent of SLOs but also functionally active as ectopic GC. There is now conclusive evidence that ELS in autoimmune diseases favor the affinity maturation of B-cells *via* an antigen-driven selection process and their differentiation to plasma cells. B cell isolated from ELS in autoimmune conditions display highly somatically hypermutated Ig VH and VL regions in line with a local antigen-driven process (45–47). Furthermore, lineage tree analysis of the Ig gene repertoire of B-cells and plasma cells infiltrating ELS + tissues in autoimmune diseases proved that clonal diversification and differentiation to antibody-producing cells take place within ELS (45, 47, 48). In keeping with the above evidence, B cells within ELS display detectable activation-induced cytidine deaminase (49), the enzyme which regulates both somatic hypermutation and isotype class switching of the Ig genes (50).

Accumulating experimental data indicate that ELS in autoimmune diseases preferentially favor affinity maturation and clonal selection toward autoantigens, which are frequently the target of autoantibodies detectable in the patients’ circulation (Figure 1B). Specifically, ELS allow the selection and differentiation of autoreactive B cells into high-affinity plasma cells reacting against citrullinated antigens in RA (49), anti-ribonucleoproteins Ro/SSA and La/SSB in SS (40), thyroglobulin and thyroperoxidase in Hashimoto’s thyroiditis (35), and insulin in type 1 diabetes (51, 52). More in details, we and other provided evidence that (i) perifollicular CD138^+^ plasma cells frequently bind biotinylated citrullinated fibrinogen and the Ro52 antigen in synovial and salivary gland ELS of RA and SS patients, respectively, but not vice versa, using double immunofluorescence experiments (40, 49, 53, 54); (ii) the engraftment of ELS + tissue from the RA synovium, SS salivary gland, and MG thymus in SCID mice in a series of chimeric human/murine models resulted in the release and detection of human autoantibodies against disease-specific autoantigens in the mouse circulation (54–56). More recently, (iii) by combining single B-cell sorting, Ig VH and VL gene cloning, and recombinant monoclonal antibody production from ELS + RA synovia or from ACPA + RA synovial fluid, we and others demonstrated that around 30% of the synovial humoral response is directed toward citrullinated antigens (36, 48).

Thus, in conclusion, although the processes underlying ELS formation in autoimmune diseases largely follow a stereotyped lymphoid neogenesis process, the autoantigens driving the autoimmune response within ELS in the respective target organs appear to be disease specific. An important consequence of this phenomenon is that a better understanding of the fine specificity of the autoantigens driving the autoimmune response within ELS would strongly enhance our knowledge of the underlying processes perpetuating autoimmunity and chronic inflammation in the different autoimmune diseases. Furthermore, and perhaps more importantly, the identification of dominant autoantigens driving B and T cell responses, as recently suggested by large throughput sequencing studies in the RA synovium, could pave the way for future vaccination and tolerogenic therapeutic strategies (57, 58).

## Author Contributions

All authors listed have made substantial, direct, and intellectual contribution to the work and approved it for publication.

## Conflict of Interest Statement

The authors declare that the research was conducted in the absence of any commercial or financial relationships that could be construed as a potential conflict of interest.
